# Visualization of the Anisotropy of the Velocity Dispersion and Characteristics of the Multi-Velocity Continuum in the Regions of Multi-Stream Flows of Gas-Dust Media with Polydisperse Dust

**DOI:** 10.3390/jimaging6090098

**Published:** 2020-09-17

**Authors:** Mikhail A. Bezborodov, Mikhail A. Eremin, Vitaly V. Korolev, Ilya G. Kovalenko, Elena V. Zhukova

**Affiliations:** Institute of Mathematics and Information Technology, Volgograd State University, Universitetskij Prospekt, 100, 400062 Volgograd, Russia; mabezborodov@rambler.ru (M.A.B.); ereminmikhail@gmail.com (M.A.E.); korolev.vv@volsu.ru (V.V.K.); zhu4ok88@mail.ru (E.V.Z.)

**Keywords:** hydrodynamic modeling, multiphase flow, polydisperse dust, visualization of flow, computational color science, cluster analysis

## Abstract

Collisionless media devoid of intrinsic stresses, for example, a dispersed phase in a multiphase medium, have a much wider variety of space-time structures and features formed in them than collisional media, for example, a carrier, gas, or liquid phase. This is a consequence of the fact that evolution in such media occurs in phase space, i.e., in a space of greater dimensions than the usual coordinate space. As a consequence, the process of the formation of features in collisionless media (clustering or vice versa, a loss of continuity) can occur primarily in the velocity space, which, in contrast to the features in the coordinate space (folds, caustics, or voids), is poorly observed directly. To identify such features, it is necessary to use visualization methods that allow us to consider, in detail, the evolution of the medium in the velocity space. This article is devoted to the development of techniques that allow visualizing the degree of anisotropy of the velocity fields of collisionless interpenetrating media. Simultaneously tracking the behavior of different fractions in such media is important, as their behavior can be significantly different. We propose three different techniques for visualizing the anisotropy of velocity fields using the example of two- and three-continuum dispersed media models. We proposed the construction of spatial distributions of eccentricity fields (scalar fields), or fields of principal directions of the velocity dispersion tensor (tensor fields). In the first case, we used some simple eccentricity functions for dispersion tensors for two fractions simultaneously, which we call surrogate entropy. In the second case, to visualize the anisotropy of the velocity fields of three fractions simultaneously, we used an ordered array (3-vector) of eccentricities for the color representation through decomposition in three basic colors. In the case of a multi-stream flow, we used cluster analysis methods to identify sections of a multi-stream flow (beams) and used glyphs to visualize the entire set of beams (vector-tensor fields).

## 1. Introduction

In astronomy, images are multidimensional data requiring visualization, three-dimensional (3D) [1], five-dimensional (5D) [2] or even six-dimensional (6D) [3], including, in one combination or another, spatial and velocity coordinates in phase space. Image resolution is increasing every year, so that each such complete set of multidimensional data can include terabytes of information. Finding physically significant features and hidden structures in multidimensional datasets is becoming an increasingly difficult task. To solve it, both automated data analysis tools and interactive tools based on data visualization are used. Traditional automated methods of working with images such as filtering, noise reduction, image contrast enhancement, etc., actually perform a preprocessing function. The main burden of understanding images falls on specialized algorithms for visualization (building histograms, graphs and trees, spectral and correlation analysis, highlighting clusters, regions and contours, tracking regular image elements, both static and dynamic), which allow for operational expert evaluation of the information content of the data and in-depth analysis of the most important part of it. In this regard, the development of new data visualization tools remains an important task. In addition, the methods for identifying features developed in the framework of working with multidimensional data can be applied in real time to the process of computer modeling, during which these data themselves are formed [4].

Examples of features of interest to researchers are, for example, caustics, which are regions of high concentration in the fields of the studied quantities. Caustics can occur in both the distribution of matter [5,6,7] and the distribution of velocities [8]. Moreover, it is curious that when analyzing 3D images with PPV coordinates (two positions on the image plane + radial velocity), heated debates are still going on as to whether these caustics are a consequence of, the kinematics [9,10] or the concentration of matter [11]. This testifies to the fact that at present there is still no generally accepted mathematical method for searching and isolating such structures, as well as an agreed point of view on the interpretation of the results obtained.

This work is devoted to the development of new methods for visualizing the flows of multiphase media with interpenetrating multi-velocity flows.

As a rule, key information on the structure of complex multidimensional flows of a continuous medium is contained in low-dimensional objects—at special points of the flow, at which abrupt changes in flow parameters occur, the continuity of the medium is disrupted, or interpenetrating flows appear.

In the case of collisional media (gases), multi-stream flows are forbidden and there are only three types of singularities, shock, tangential and contact discontinuities. For weakly collisional or collisionless media, the existence of interpenetrating flows is typical, as a result of which a noticeably greater variety of specific types of features can arise, such as turning, stagnation and accumulation points, multi-stream areas, collisionless shock waves [12], folds, sheets and caustics [13]. The combined case, in which the fluid includes both collisional and collisionless components, is even richer in various manifestations of singularities. An example of this kind of media is multiphase media such as gas suspensions or dusty gases.

In the case of collisionless component of a special type, namely, impurities of small solid particles immersed in the carrier gas phase (collisional component), the presence of strong friction between the solid particles and the gas is assumed, and the fluctuation velocities of inclusions and, accordingly, their own stresses are usually neglected.

If we consider the finite inertia of inclusions and the non-vanishingly small value of fluctuation velocities, then it should be noted that one of the distinctive dynamic features of the dispersed phase is the ability of the particle velocity dispersion to be anisotropic. In contrast to this, in gas dynamics, the velocity dispersion of a continuous medium is characterized not by a tensor, but by a single number, the temperature, which implies the conservation of isotropy in the velocity distribution of gas particles at any point in the medium at any time.

There is a widespread point of view according to which the motion of the impurity phase can be simulated either by assuming an impurity completely “frozen in” into the carrier phase, or by considering the impurity particles as low-inertia, so that their deviation from the streamlines of the carrier phase is insignificant. Often in such cases the Kramers-Fokker-Planck equation [14] is used, which implies the prevalence of the friction force over all other forces acting on the particles.

Under conditions when external volumetric or surface forces act on impurity particles, as, for example, in the problem of dynamic equilibrium of a gravitating astrophysical medium, these external forces can significantly exceed the friction force. However, even if there are no other forces acting on the impurity particle in the medium, apart from the friction force, in the case of sufficiently inertial particles, the fulfillment of the conditions of the Kramers-Fokker-Planck approximation is by no means guaranteed.

The mismatch of the motions of the dispersed and carrier phases occurs in shock waves with partial dispersion, in which the parameters of the flow of the carrier phase undergo a discontinuity at the wave front, and the parameters of the dispersed phase are continuous. In the regions behind the fronts of such shock waves, relaxation of the flow parameters of the dispersed phase occurs.

Conversely, the passage of a sequence of shock fronts can result in a multi-stream flow of an impurity medium with the intersection of trajectories. A specific mismatch between the motions of the impurity and the carrier phases is also possible, such that it leads to the appearance of smoothed shock transitions in which there is no hydrodynamic discontinuity in the carrier phase for the so-called waves with total dispersion [15]. Finally, flows can exist without any singular concentration behavior, but those with significant relative flows of streams do not simply exist, but play an important role in the dynamics of themedium. For example, if the relative motion of flows in a collisionless plasma occurs at a speed exceeding the thermal one, a two-beam instability [12] develops in the medium.

In recent years, great interest has arisen in the study and, accordingly, the numerical modeling of the dynamics of polydisperse mixtures [16]. The behavior of individual fractions of dust particles makes it possible to diagnose the state of the carrier phase. For example, in astrophysics, shock waves in rarefied interstellar gas are often impossible to observe directly, as gas almost does not cause extinction of light, while the extinction on dust is large.

A convenient qualitative tool for analyzing the flows of a gas-dust medium is visualization of the areas of localization of structural features of the flow, caustics, folds, etc. Special techniques are being developed for describing the behavior of a dispersed admixture in flows with discontinuities, multiple intersections of particle trajectories, and local zones of accumulation of the dispersed phase using additional equations that highlight the features [17,18]. However, to describe flows in which there are many multi-flow areas (in the limit, the entire computational domain is a multi-stream flow), it is simpler and more economical to directly simulate the dynamics of the impurity phase using the particle method.

The visualization of flows of complex media faces difficulties associated with limited resources for visualization.

For example, within the framework of the gas dynamics of a monophase medium dealing with collisional media (gas and liquid), it is often sufficient to restrict ourselves to a simplified representation of a continuous medium (compressible or incompressible) as a perfect fluid. By definition, there are no tangential stresses in a perfect fluid, and then the dynamics of a perfect fluid can be determined using only scalar (temperature, concentration, and pressure) and vector (mass and velocity) functions of coordinates and time.

Non-perfect fluids are not locally isotropic. To describe them, it is also required to specify tensor field characteristics, for example, the velocity dispersion tensor. For the visualization of fields of different mathematical nature, various visualization tools are applicable. The simplest means of visualizing a scalar field can be color; for visualizing a vector field, a set of oriented segments (arrows); for visualizing a tensor field, a combination of the above, as well as glyphs [19,20,21].

If the medium has a complex structure, for example, the medium is multiphase and is a multi-velocity continuum, then we have to consider the idea of a multi-field description of the state of the medium. The visualization of multi-field distributions has limited resources. As there are only three basic colors in all colorimetric systems, up to three scalar fields can be visualized simultaneously in one drawing, restoring their values as coefficients of decomposition of an arbitrary color into three basic components [22,23]. On the other hand, for visualization, geometrical objects (glyphs) of any complex structure can be used, and these can be information-intensive, but difficult to perceive. Thus, a compromise must be found between increasing the information content of visualization tools and clarity.

An example of a successful combination of visualization tools for fields of different nature are renzograms used in astronomy for the simultaneous visualization of concentration or emission fields (scalar fields) and velocities (vector fields) in one image [11,24]. More refined methods are also proposed that use directly the resources of the GPU itself, such as transfer functions and graphics shaders, and allow combining spatial and kinematic data in one multidimensional image [1]. In fact, this is another direction in the development of high-performance data processing methods based on the computational capabilities of graphic cards, in which the speed of calculations and data transfer grows with each new generation.

In this work, we propose three approaches to visualizing flows in a two-phase gas-dust medium containing dust particles of various types (a polydisperse dust mixture). The aim is to construct methods for convenient (i.e., both informative and simple) visualization of the spatial distribution of the anisotropy of the velocity dispersion for particles of several different fractions simultaneously, as well as visualization of the kinematic characteristics of particle fluxes in regions of multi-stream flow.

Consideration was performed from the simplest method using a specially selected entropy measure to visualize the degree of anisotropy of the velocity dispersion of two fractions at once, to a combined method using visualization techniques using glyphs and cluster analysis methods to identify individual particle beams in the velocity field in the areas of multi-stream flow.

## 2. Numerical Model of the Flow of the Interstellar Gas and Dust Medium in the Galactic Disk

We developed visualization tools using the example of a two-dimensional dynamic model of a turbulent interstellar gas-dust matter passing through the spiral arm of a disk galaxy. Details of modeling, basic equations, and a justification of the applicability of certain approximations can be found in our work [22]. Unlike the model presented in [22], here, we are considering not a horizontal, but a vertical section of the spiral arm and its galactic surroundings. Here, we will only briefly list the main features of the model.

**Figure 1:**
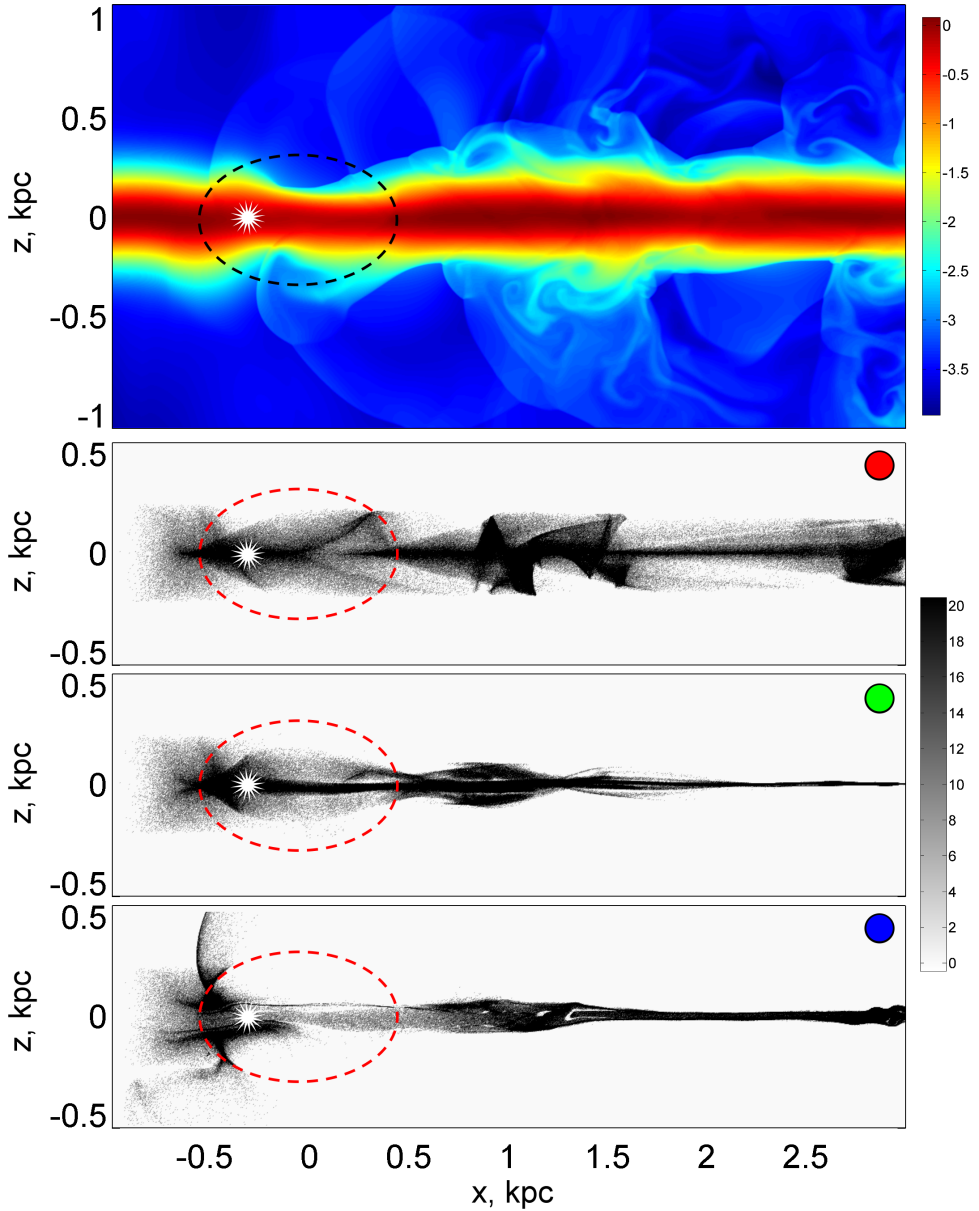
Distributions of the gas density (logarithmic scale), normalized to the initial value of the density $\rho_{0}$, in the gas disk of the galaxy (top color figure) and concentration (in the number of particles per computational cell) for the fractions of large (figure with a red marker), medium (figure with a green marker), and small (figure with a blue marker) dust particles. The dashed ellipse shows the contour of the spiral arm, and the asterisk indicates the position of the radiation source.

**Figure 2:**
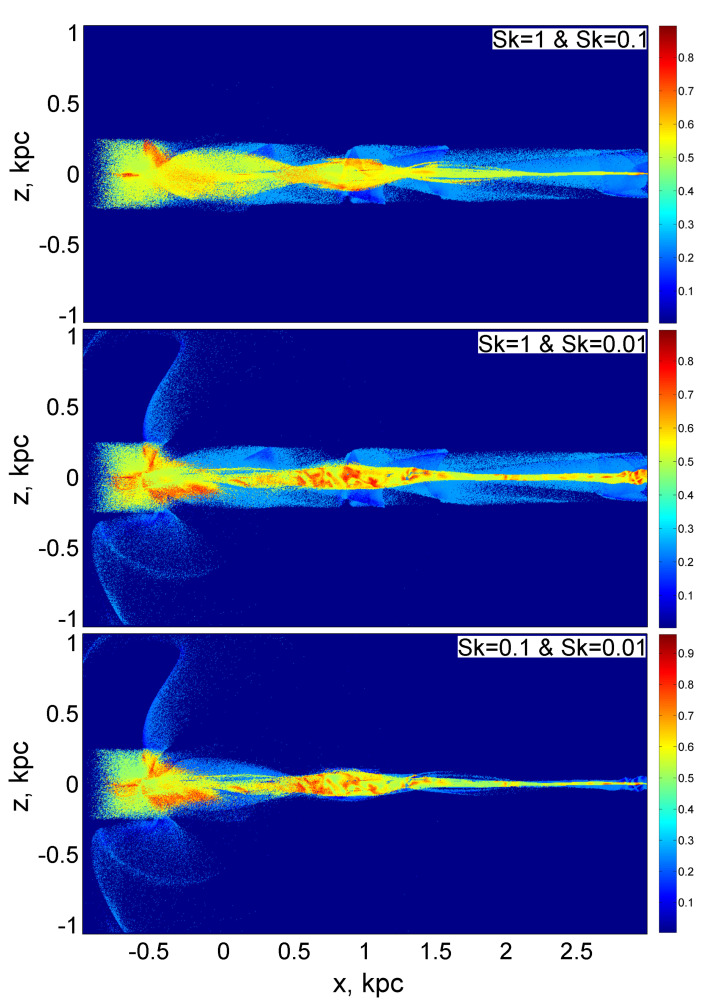
Distributions of the surrogate entropy for a mixture of particles of coarse and medium fractions (upper figure), a mixture of particles of coarse and fine fractions (middle figure), and a mixture of medium and fine particle fractions (lower figure).

**Figure 3:**
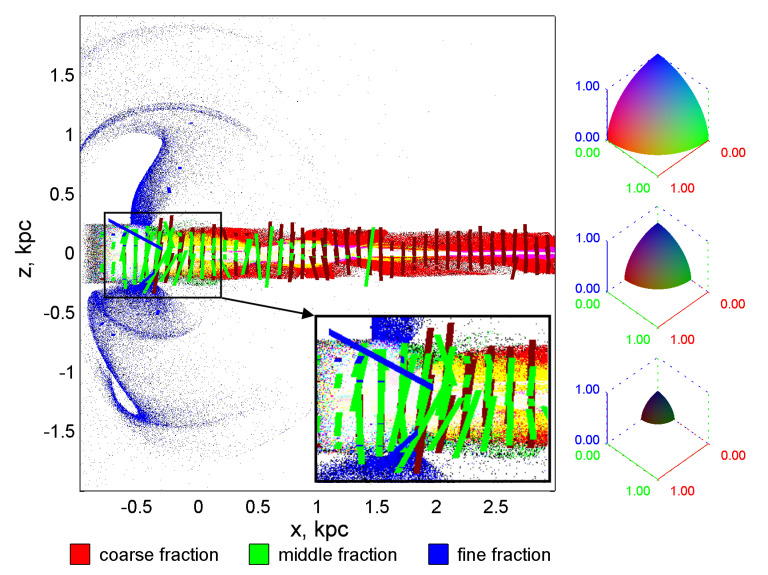
The distribution of eccentricities (scalar color field) and tensor field of the main directions of dispersion ellipses for a mixture of three particle fractions. The segments represent the part of the tensor field corresponding to those principal directions along which the dispersion is maximum.

(i) A vertical section of a disk galaxy is considered. The thickness (in the longitudinal direction along the disk, that is, along the *x* axis) of the spiral arm equal to 1 kiloparsec is taken as the characteristic spatial scale *L* of the problem. All lengths are expressed in units of a given characteristic spatial scale (in kpc). In Figure 1, which presents the snapshots of gas (upper panel) and dust (underlying panels) concentrations, the spiral arm is marked with a dashed line. The vertical section of the arm has an elliptical profile with a vertical, minor semiaxis of 0.35 kpc. The half-thickness of the galactic disk is 0.25 kpc.
  The lateral dimensions of the disk and the arm are determined by the gravitational potentials, which have a one-dimensional Gaussian profile for the disk along the vertical coordinate *z* and, accordingly, a two-dimensional, elliptical Gaussian profile for the arm. The depth of the disk potential, expressed in units of the square of the initial, determined at the input left boundary, speed of sound in the gas $c_{s0}^{2}$, is 5, and the depth of the spiral arm is 1. We assumed that the arm extends along an axis transverse to the plane of the figure and its curvature along this direction can be neglected; therefore, the flow pattern in the first approximation can be considered as two-dimensional, unfolding in the (x,z) plane. Outside the galactic disk, the density of gas is extremely low; this region is called the galactic halo.
(ii) The flow is considered in the computational domain, which has the shape of a square. The number of grid cells is equal to $N_{x}\times N_{z}$, where $N_{x}=N_{z}=1024$.
(iii) The carrier phase is considered as a collisional monatomic perfect gas. The gas flow is described as adiabatic. In the calculations, the gas adiabatic exponent was taken equal to 5/3. Gas flows into the computational domain from the left boundary and passing through the region inside and in the vicinity of the spiral arm, including flowing around it from above and below, freely flow out through the right boundary.
(iv) The dispersed phase is considered as a passive scalar impurity. The dynamics of individual impurity particles (dust grains) are calculated by direct numerical simulation using the particle method.
(v) Dust is considered as a collisionless environment in relation to itself. This assumption is justified by the fact that the concentration of dust in galaxies is significantly lower than the concentration of gas, and the interparticle distances for dust grains in the interstellar medium are large. On the other hand, the interaction of dust with gas is considered through the influence of the friction force of dust grains on the gas. The reverse effect of dust on the gas is not taken into account, which is justified by the fact that the mass of dust in galaxies is small in comparison with the total mass of gas. The possibility of dust having a high velocity in relation to gas is taken into account. The friction force depends on the relative velocity of the dust particles as follows.
  At low, substantially subsonic speeds, the Stokes friction regime is realized (linear dependence of the friction coefficient on the speed). If the relative speed is comparable to or exceeds the speed of sound in a gas, the friction coefficient acquires a quadratic dependence on the speed. Dust can acquire a supersonic relative velocity behind shock jumps in gas. In the interval of intermediate values of velocities, the friction coefficient continuously sews both asymptotics, subsonic and supersonic.
(vi) The dust component is considered as a polydisperse mixture of three different fractions. The corresponding Stokes numbers (recall that the Stokes number characterizes the relative time of dynamic relaxation of particles due to viscous friction) are equal for large particles Sk=1, for medium-sized particles Sk=0.1, and for small particles Sk=0.01, which correspond to the radius of the dust grains in 0.03, 0.3, and 3 μm, respectively. The dimensionless Stokes number characterizes the degree of dynamic connectivity of the gas and impurity particles through friction. This is formally defined as the ratio of the dynamic relaxation time $\tau_{fr}$ to the characteristic dynamic time τ of the problem:
  (1) $$\mathrm{Sk}=\frac{\tau_{fr}}{\tau }.$$
  The dynamic is the time taken for sound ($c_{s0}=10$ km/s for the sound speed for the warm interstellar gas) to travel on a characteristic spatial scale L=1 kpc.
(vii) The maximum number of particles for each fraction is limited from above by the number $3.5\times 10^{6}$, which corresponds to the fractional participation of 3.3 particles of each fraction in each calculation cell on average. The typical average number of particles of a single fraction was $1.5\times 10^{6}$.
(viii) The number of particles in the computational domain was set to be variable. In a time equal to 3 time units, $3.5\times 10^{6}$ particles are injected into the computational domain from the left boundary in the range of height from z=−0.25 to 0.25 kpc and length from x=−1 to −0.75 kpc. The particles are carried by the gas flow and, reaching the right boundary, are carried along with the gas outside the computational domain. For the gas on the right boundary, free boundary conditions are used.
(ix) Turbulence is generated by random forces. In the implementation of the turbulence generation procedure, we follow the work [25]. Dust grains of the fraction of large particles are more strongly clustered than representatives of other fractions and clearly outline the periphery of turbulent vortex cells.
(x) In a circular region with a radius of 10 computational cells centered at a point with a coordinate (x=−0.3 kpc, z=0), there is a source of powerful radiation, which is understood as young stars born behind the front of a galactic shock wave in the area of sharp gas compression that promotes rapid star formation. Such rows of young stars are clearly observed in the arms of spiral galaxies, as extended bright regions located behind dust lanes stretching along the inner edge of the arms [26,27,28].
  The introduction of a light source into the model makes it possible to simulate the concentration of dust at the entrance to the spiral arm, since powerful radiation retains a significant part of the dust in front of the radiation source and does not allow the dust to be immediately carried away by the gas flow. The gradual drift of dust occurs due to turbulence, which tends to destroy the dust lanes. Due to the two-dimensional geometry of the model, the radiation source is assumed to be infinitely extended in the direction perpendicular to the plane (x,z); therefore, the radiation intensity is specified as a quantity that weakens in inverse proportion to the distance from the source.
(xi) Both dust and gas experience the action of a gravitational force that tends to return matter from large galactic heights z≠0 to the equatorial plane of the disk z=0. For dust, especially for the fraction of large dust grains, this motion looks like an alternation along the *x* axis of the oncoming motion of converging dust streams or, conversely, the motion of streams diverging from the galactic plane. In the halo, the force of gravity is weak, the friction force is also small, as the gas in this region is highly rarefied, therefore, the radiation pressure force serves as any significant force for the dust grains. Figure 2 and Figure 3 illustrate the emission of fine dust at great heights above the plane of the disk.

## 3. Measures of Anisotropy of the Velocity Field

All the values described in this section are calculated individually for each fraction.

Let there be an *N*-dimensional vector field of velocities $v_{a}$ of dust particles located in the *N*-dimensional configuration space. The spatial coordinates of any *a*-th particle are $v_{a}$. Let also a computational grid ($N_{1}\times \ldots \times N_{N}$) be given in this configuration space, and the discrete coordinates of each grid cell are $\left(i_{1},\;\ldots ,\;i_{N}\right)$.

We find the mass velocity V of the dust component in a cell of the computational domain with coordinates $\left(i_{1},\;\ldots ,\;i_{N}\right)$, summing over the velocities of all particles in this cell:

(2) $$V\left(i_{1},\;\ldots ,\;i_{N}\right)=\frac{1}{N_{d}\left(i_{1},\;\ldots ,\;i_{N}\right)}\underset{x_{a}\in \left(i_{1},\;\ldots ,\;i_{N}\right)}{\sum_{a}}v_{a}\left(x_{a}\right),\;i_{1}=\bar{1\ldots N_{1}},\;\;\ldots ,\;\;i_{N}=\bar{1\ldots N_{N}}.$$

Here, $N_{d}\left(i_{1},\;\ldots ,\;i_{N}\right)$ is the number of particles in the cell with coordinates $\left(i_{1},\;\ldots ,\;i_{N}\right)$. The calculations are performed for those cells in which $N_{d}\left(i_{1},\;\ldots ,\;i_{N}\right)\geq 1$. In empty cells, all mean values and variances are assumed to be zero. (In real calculations, we depicted the dispersion values as nonzero only for cells with $N_{d}\left(i_{1},\;\ldots ,\;i_{N}\right)>3$ to avoid the influence of large statistical errors.)

Next, we determine the fluctuation velocities $u_{a}$ of particles

(3) $$u_{a}\left(x_{a}\right)=v_{a}\left(x_{a}\right)-V\left(i_{1},\;\ldots ,\;i_{N}\right),\;x_{a}\in \left(i_{1},\;\ldots ,\;i_{N}\right),\;i_{1}=\bar{1\ldots N_{1}},\;\;\ldots ,\;\;i_{N}=\bar{1\ldots N_{N}}.$$

The velocity dispersion tensor is defined as symmetric, positive, and semi-definite

(4) $$D\left(i_{1},\;\ldots ,\;i_{N}\right)=\frac{1}{N_{d}\left(i_{1},\;\ldots ,\;i_{N}\right)}\sum_{a}u_{a}u_{a}^{T}.$$

Therefore, it has *N* real non-negative eigenvalues (principal values) $\sigma_{1}^{2}\left(i_{1},\;\ldots ,\;i_{N}\right)$, …, $\sigma_{N}^{2}\left(i_{1},\;\ldots ,\;i_{N}\right)$. The quantities $\sigma_{i}^{2}$ are called velocity dispersions. These quantities are invariants of the dispersion tensor (scalars). We can say that the tensor is associated with the *N*-dimensional multi-scalar $\left\{\sigma_{1},\;\ldots ,\;\sigma_{N}\right\}$.

The tensor (4) is also associated with its principal directions given by its eigenvectors, which constitute an orthonormal basis in $R^{N}$: $\left\{n_{1},\;\ldots ,\;n_{N}\right\}$. The dispersion tensor can then be represented in terms of the eigenvectors and eigenvectors in the form

(5) $$D\left(i_{1},\;\ldots ,\;i_{N}\right)=\overset{N}{\sum_{i=1}}\sigma_{i}^{2}n_{i}n_{i}^{T}.$$

In what follows, for definiteness, let the eigenvalues be ordered in the direction of non-increasing values: $\sigma_{1}^{2}\geq \sigma_{2}^{2}\geq \ldots \geq \sigma_{N}^{2}$.

With a symmetric, positive, semi-definite tensor, one can associate an ellipsoid whose axes directions coincide with the principal directions, and the inverse squares of the semiaxis lengths are equal to the corresponding dispersions. The measure of the oblateness of the ellipsoid in the plane ($x_{i},x_{j}$) is the eccentricity

(6) $$e\left(i_{1},\;\ldots ,\;i_{N}\right)=\sqrt{1-\frac{\sigma_{j}^{2}\left(i_{1},\;\ldots ,\;i_{N}\right)}{\sigma_{i}^{2}\left(i_{1},\;\ldots ,\;i_{N}\right)}}.$$

We assumed here that $\sigma_{j}^{2}\leq \sigma_{i}^{2}$. The isotropic distribution of the velocity dispersion corresponds to the value e=0. For completely anisotropic, e=1.

In astronomical literature, the dispersion of velocities is often understood as a quantity with the dimension of velocity and, in its meaning, is equal to the standard deviation (SD). Therefore, we also introduce the SD-tensor

(7) $$S\left(i_{1},\;\ldots ,\;i_{N}\right)=D^{1/2}\left(i_{1},\;\ldots ,\;i_{N}\right)=\overset{N}{\sum_{i=1}}\sigma_{i}n_{i}n_{i}^{T}.$$

The eigenvalues $\sigma_{i}$ are called standard deviations.

## 4. Geometrical Primitives for Visualizing Anisotropic Velocity Distribution

By geometric primitives, we mean a basic set of geometric shapes that underlie all graphic constructions. If we want to visualize the tensor field of velocity standard deviations in velocity space, then, in accordance with the conclusions of the previous section, we should use an ellipsoid as a key geometric figure.

The equation for the set of points that completely fills the *N*-dimensional ellipsoid has the form

(8) $$v=V+\sum_{i=1}^{N}\sigma_{i}n_{i}\left(n_{i}\cdot \xi \right),\;\xi \in R^{N},\;\left|\xi \right|\leq 1.$$

If we want to restrict ourselves to the image of only the surface of the ellipsoid, then the inequality in (8) must be replaced by an equality.

In the particular case N=2, the ellipsoid turns into an oriented ellipse; in the case N=1, into an oriented segment; and for N=0, into a point.

## 5. Visualization of the Anisotropy Distribution of the Velocity Dispersion of Polydispersed Dust. Scalar Multi-Fields: 2 in 1

We consider two-dimensional distributions (N=2). To visualize the distributions of the anisotropies of the velocity dispersions for several fractions simultaneously in one figure, we propose, in the spirit of our work [22], to represent these distributions using a certain indicator (function of object features) characterizing the degree of diversity of the distribution of features in the sample. Shannon’s entropy is often taken as a measure of diversity.

In this paper, we propose a modification of the Shannon entropy, which we call *surrogate entropy*. Consider an algorithm for calculating the entropy for the case of a mixture of two fractions. We define the surrogate entropy according to the following rule:

(9) $$H=\left\{\begin{array}{cc} -\sum_{\alpha =1}^{2}\sum_{i=1}^{2}p_{i}^{\left(\alpha \right)}\log_{4}\left(p_{i}^{\left(\alpha \right)}\right), & \mathrm{both}\;\mathrm{fractions}\;\mathrm{in}\;\mathrm{the}\;\mathrm{cell};\\ \frac{1}{2}+\sum_{i=1}^{2}p_{i}^{\left(1\right)}\log_{4}\left(p_{i}^{\left(1\right)}\right), & \mathrm{only}\;\mathrm{one}\;\mathrm{fraction}\;\mathrm{in}\;\mathrm{the}\;\mathrm{cell};\\ 0, & \mathrm{no}\;\mathrm{fraction}\;\mathrm{in}\;\mathrm{the}\;\mathrm{cell}. \end{array}\right.$$

Here, the index α marks the corresponding fraction, the index *i* is the ordinal number of the principal axis.

The values of $p_{i}^{\left(\alpha \right)}$ in (9) are determined as the relative inverse squares of the lengths of the semiaxes of the dispersion ellipse for each fraction separately in accordance with the formula

(10) $$p_{i}^{\left(\alpha \right)}=\left\{\begin{array}{cc} \frac{{\left(\sigma_{i}^{\left(\alpha \right)}\right)}^{2}}{N_{f}\left({\left(\sigma_{1}^{\left(\alpha \right)}\right)}^{2}+{\left(\sigma_{2}^{\left(\alpha \right)}\right)}^{2}\right)}, & \mathrm{if}\;\mathrm{the}\;\mathrm{fraction}\;\mathrm{in}\;\mathrm{the}\;\mathrm{cell}\;\mathrm{is}\;\mathrm{presented};\\ 0, & \mathrm{if}\;\mathrm{the}\;\mathrm{fraction}\;\mathrm{is}\;\mathrm{not}\;\mathrm{represented}\;\mathrm{in}\;\mathrm{the}\;\mathrm{cell}. \end{array}\right.$$

Here, $N_{f}$ is the number of fractions represented by their particles in the cell. For example, if particles of both fractions are present in a cell, then $N_{f}=2$, if only one, then $N_{f}=1$. If the cell is free of dust, then division by $N_{f}$ is not performed, all four values of $p_{i}^{\left(\alpha \right)}$ are set equal to zero in accordance with the definition of (10).

With this approach, the case where both eccentricities for both fractions are close to zero will hardly distinguish between situations where the eccentricities are slightly different from zero. These cases correspond to an equally weighted mixture with a maximum entropy equal to $-\log_{4}\left(1/4\right)=1$, which is displayed in dark red.

A medium, green color indicates a combination in which the velocity dispersions of both fractions are completely anisotropic. Then, the surrogate entropy is $-\log_{4}\left(1/2\right)=0.5$. The same green color marks the case when one of the fractions is absent, and the velocity dispersion of the second fraction is completely anisotropic. The entropy is again equal to $0.5+\log_{4}\left(1\right)=0.5$.

In the case when only one fraction is present in the cell and its velocity dispersion is completely isotropic, the entropy takes the minimum possible value within the framework of this problem, equal to $0.5+\log_{4}\left(1/2\right)=0$. Dark blue is assigned to this value of entropy. The same dark blue is mapped to empty dust-free cells. The distributions of surrogate entropy for all possible pairwise combinations of three fractions are shown in Figure 2.

The advantage of visualizing spatial distributions using entropy is that it can be used for monochrome (black and white) printing, although, in our example, we used a multi-colored Jet colormap for greater clarity.

## 6. Visualization of the Anisotropy Distribution of the Velocity Dispersion of Polydisperse Dust. Scalar-Tensor Multi-Fields: 3 in 1

Unfortunately, it is not possible to generalize the surrogate entropy to the case of three or more fractions. Therefore, we propose to use the decomposition on basic colors to visualize the anisotropy of the velocity distribution of three fractions at once, instead of entropy. This approach is applicable to visualize the simultaneous distribution of three, two, and one fractions. In the first two cases, color printing is inevitable.

The color in the cell is determined by the [r,g,b] palette, in which the shade of each of the three color channels is equal to the eccentricity of the ellipse for the given fraction, large (red), medium (green), and small (blue), respectively (Figure 3). In particular, if all eccentricities are 0, the cell color is black, and if all eccentricities are 1, then the color is white. The contribution to the total color is zero for those fractions, the number of dust grains of which in the cell is less than three. The cell is also colored white if the cell contains less than three particles for all factions.

We attempted to make the image more informative; therefore, we supplemented the scalar field of the distribution of eccentricities with the tensor field of dispersions, more precisely, with that part of it that corresponds to the “most principal” direction, namely the largest axis of the dispersion ellipse.

The segments are plotted in every twenty-fifth cell in the *x*-, and in *z*-directions for each of the three fractions. Their color symbolizes this or that faction. The dark red lines represent the coarse fraction, the green ones represent the middle ones, and the blue ones represent the fine ones. Mathematically, the segment that visualizes the major axis of the ellipse is given by the Equation (8), which takes N=1. The length of each segment is given in relative units and is proportional to the local value of the dispersion along the corresponding principal direction. The lengths of the segments are selected so that the global maximum of the dispersion in the entire computational domain, which corresponds to a segment length of 0.5 (kpc).

It is clearly seen from Figure 3 that the main spread in velocities is concentrated in the vertical direction. The particles oscillate near the bottom of the gravitational pit of the galactic disk in this direction.

In addition, the fraction of small particles relaxes most rapidly to dynamic equilibrium, followed by the relaxation of medium particles, and large particles do not have time to relax due to their inertia throughout the entire length of the disk in the computational domain.

## 7. Visualisation of Multi-Stream Flows. Vector and Vector-Tensor Multi-Fields: Many in 1

The separation of particles into fractions can be due to numerous reasons: differences in mass, size, shape, chemical composition, the optical properties of particles, etc. In the previous sections, we dealt with fractions, the number of which was fixed and the division into which was already predefined before the start of the simulation.

A nontrivial task is to visualize a polydisperse medium, in which the number of fractions is variable and changes from point to point and from one point in time to another.

We can think of separate clusters of particles (beams) as forming or disappearing fractions. Each separate beam is characterized by the fact that its constituent particles have a similar velocity value and these velocities differ markedly from the velocities of other particles of the medium. The flow breaks up into such separate beams if the particle trajectories begin to intersect. In this case, the visualization procedure must be adapted to the search and detection of individual beams and to their adequate visualization.

There are various techniques for finding areas of multi-stream flow. For example, in 3-dimensional computer cosmological modeling of expanding cold dark matter in the Universe, regions of multi-flow are associated with areas of high density of matter, caustics. Caustics act as gravitational wells decelerating or capturing particles of matter; therefore, the boundaries of multi-flow regions are identified as turning points of particle trajectories (according to [4], flip-flops of fluid particles). However, such an approach is not applicable in the case of gas and dust flows considered in our article, when the dynamics of a collisionless admixture is not related to its own gravity, but mainly depends on the motion of the carrier medium.

It is convenient to analyze the kinematic properties of a multiphase gas and dust flow using geometric methods.

The states of particle motion can be represented by a set of image points in the velocity space. The set of image points can have arbitrarily complex geometric and topological properties. However, we prefer to use a unifying approach that reduces all possible variety of objects to one single class of objects, particle beams.

By the image of a beam in the velocity space, we mean a set of points characterized by its central point (mean beam velocity) and scatter of points in space (velocity dispersion). Any configuration of beams can be considered as a family of beams embedded in a beam with the highest velocity dispersion. This representation includes, among other things, the limiting special case, when the beam covering all the other beams degenerates into an empty set of points. The set of points, enclosing all other beams, empty or non-empty, we call the background. From the point of view of cluster analysis, the background is interpreted as noise. A beam stands out relative to the background if it is a group of points with a higher concentration than the background concentration, which corresponds to the correlated velocities of particles in the beam.

A multi-stream flow can include several beams. Particle beams are separable relative to each other if their mean velocities exceed the sum of their velocity dispersions. The specific task is to recognize individual groups of particles with similar velocities in the velocity space of a large ensemble of particles. The procedure for dividing a set of points into groups is implemented by cluster analysis.

### 7.1. Cluster Analysis for Beam Identification

In the general case, the clustering problem is reduced to dividing the initial sample into a relatively small number of groups (clusters). Grouping is performed according to a given criterion so that the elements of one cluster are as similar as possible, and the elements from different clusters differ significantly. In this case, the number of groups may not be known in advance.

When segmenting the flow characteristics with a polydisperse admixture, it is necessary to process a large amount of data in proportion to the number of particles, in our case, $10^{6}÷10^{7}$. In addition, not only is the number of cluster beams unknown but also a priori information regarding their geometric and statistical properties is also lacking. Initially, it is also unknown whether there is noise in the studied data.

There are a large number of different clustering algorithms and their modifications, which are devoted to monographs [29,30,31,32,33,34,35], articles, and reviews [36,37,38,39]. The above features of the problem impose restrictions on the choice of solution methods. In particular, the algorithm should use a minimum amount of a priori data and allow the selection of clusters of different structures, shapes, sizes, and densities.

One of the most common algorithms in applications, the relatively simple k-means algorithm has a number of significant drawbacks [32]. First, before splitting data set into clusters, it is necessary to set as a parameter the number of clusters, which, in the case of a multi-stream flow, is not known in advance. However, there is a procedure for determining the optimal number of clusters [40]. According to this technique, it is required to maximize the value of the “transformed distortion”, a special functional, which is a measure of the within-cluster dispersion, from the number of selected clusters.

The algorithm suggests choosing the number at which the increase in distortion is maximum as the optimal number of clusters; this increase in the value of the distortion should have the form of a jump. A jump signals that a further increase in the number of clusters introduces an insignificant improvement in the quality of the fit. However, as the experience of our calculations shows, the jump is not always expressed clearly enough to be unambiguously identified. In addition, obtaining the dependence of the variance on the number of clusters is a computationally expensive procedure.

An alternative approach is to pre-analyze the original data set and, according to some criterion (for example, assessing cluster tendency using the Hopkins statistics [41]), determine does it allow for clustering. Further, if clustering is acceptable, the set of points should be divided by the k-means method into two parts, which should then be analyzed again for the possibility of clustering. The resulting clusters, if further clustering is possible, should be again divided into two parts and so continue this procedure until division is no longer possible.

However, no matter how the “true” number of clusters is selected, the k-means method shows its main drawback: it is poorly suited for detecting clusters of complex shapes. The selected clusters often do not correspond to the morphology of the distribution features of the points. For example, a solid strip or ring-shaped structures, contrary to common sense, are divided into clusters.

**Figure 4:**
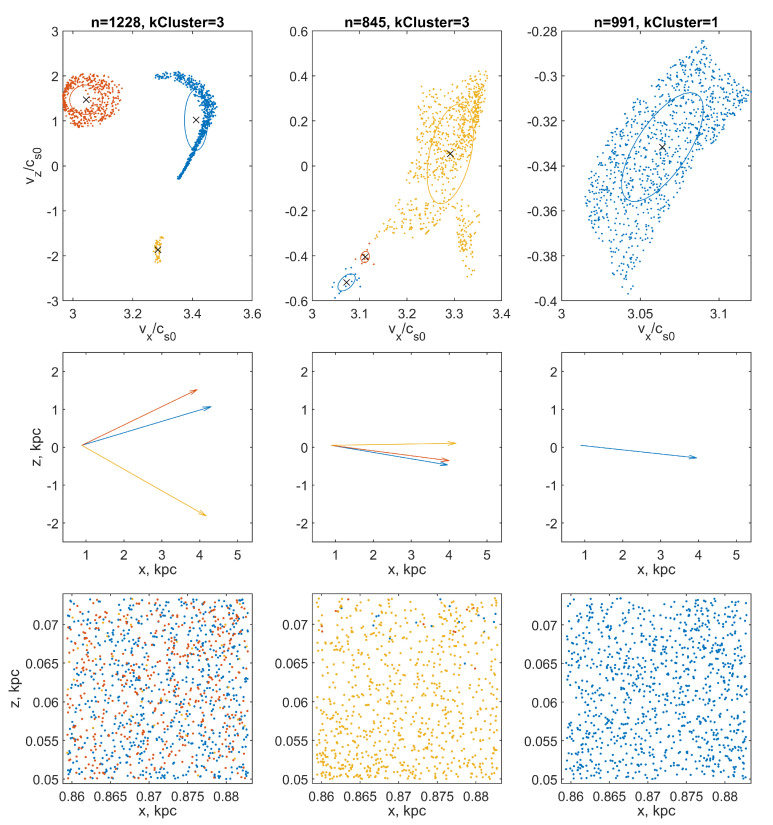
The results of cluster analysis of distributions of points representing particle velocities in velocity space. The points are grouped into clusters using the DBSCAN procedure. The color of the dots indicates the belonging of a drawing element (point, vector, or ellipse) to a specific cluster (particle beam). Top row: selected clusters of points (*n* is the total number of points in the computational domain, kCluster is the number of found clusters) in the velocity space. The × markers indicate the cluster midpoint (which corresponds to the mean beam velocity). Ellipses show cluster standard deviation ellipses. Middle row: vectors of mean beam velocities, collected in a quiver of vectors. Bottom row: the distribution of particles of each of the fractions in the coordinate space: the left column is the particles of the coarse fraction; in the middle column, the middle fraction; and in the right, the fine fraction. The analysis was performed for a cell of the computational grid with coordinates (x=0.887 kpc, z=0.05 kpc).

**Figure 5:**
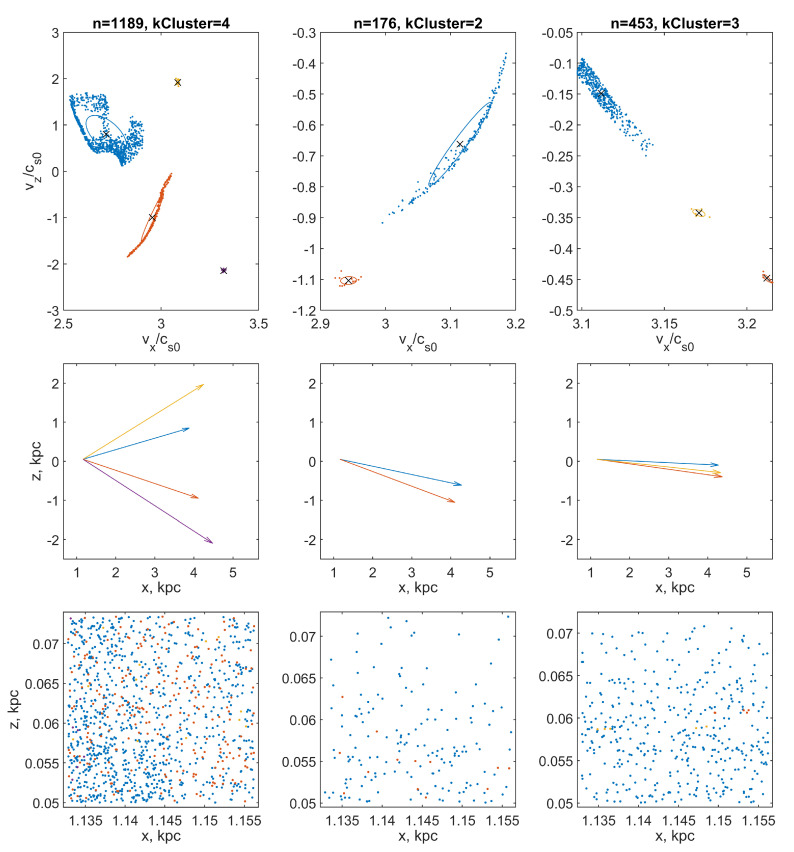
The same as in Figure 4, but for a cell with coordinates (x=1.160 kpc, z=0.05 kpc).

A significantly better quality of beam identification can be obtained using the parametric method DBSCAN [42]. Within the framework of this method, the number of clusters is determined automatically, and the parameters are the minimum number of points-neighbors minPts, on which the noise threshold depends, and the maximum radius of neighbor reachability ϵ, which characterizes the distribution density of points in a connected cluster. The selection of the optimal values of these parameters for our calculations was carried out empirically. We found experimentally that the DBSCAN clustering procedure satisfied all of the above requirements if the parameter minPts=5 for all types of particles, while the parameter ϵ=0.1 for the fraction of large particles, ϵ=0.03 for medium, and ϵ=0.01 for small particles. With such a set of parameters, the selected groups of points form connected regions without excessive clustering (Figure 4 and Figure 5, upper panels). We found the results obtained were quite satisfactory, although more refined versions of the DBSCAN algorithm are available, such as the OPTICS, HDBSCAN, and GDBSCAN [43] methods.

### 7.2. Construction of Multi-Vector and Multi-Glyph Fields Visualizing Multi-Stream Flow

**Figure 6:**
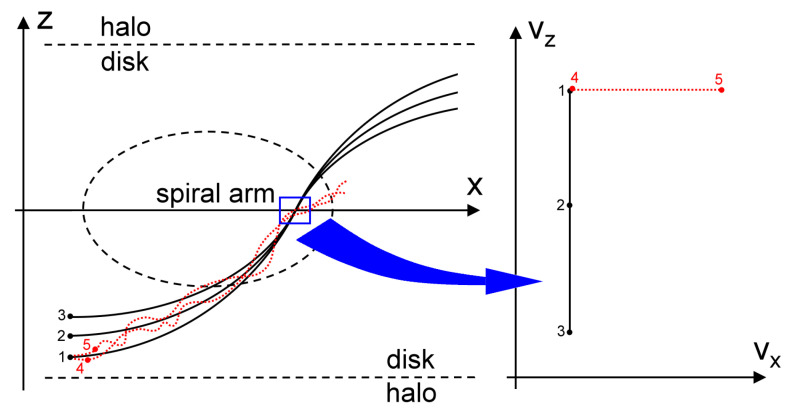
A schematic representation of the formation of quasi-one-dimensional structures in the velocity space for large particles. Left: trajectories of particles in a gas disk converging into the same cell. Right: the states of motion of the same particles in this cell.

Calculations show that at a sufficiently large distance from the entrance boundary, small particles, having time to quickly relax to velocities close to the gas velocity, quasi-uniformly fill the velocity space and no beams are traced in their velocity distribution. On the contrary, large particles tend to form bizarre quasi-one-dimensional structures in the velocity space (Figure 4 and Figure 5, upper panels). The reason for this phenomenon is explained by the schematic diagram in Figure 6.

Particles 1, 2, and 3 move mainly under the action of the gravitational force of the disk and have the same initial velocity along the disk at the initial point. As the profile of the gravitational potential in the disk for |z|<0.25 kpc is close to quadratic, the motion of the particles is close to harmonic, which is characterized by isochronism of the motions. With such a motion oscillating along the vertical axis, there will be no phase progression for particles with different vibration amplitudes. In other words, particles vibrating in the same phase will arrive at the node simultaneously, but with different vertical velocities $v_{z}$ depending on the amplitude, while their longitudinal velocities $v_{x}$ will be approximately the same. These particles correspond to a vertical segment 1-2-3 on the velocity plane. Particles 4 and 5 move not only under the influence of gravity but also are slightly carried away by the turbulent gas flow. When passing through the cell, they can have the same vertical speed, but different horizontal speeds. The depicting points of their velocities lie on the segment 4-5.

We propose to use both simple and composite glyphs to simultaneously visualize multiple vector and tensor fields that specify the direction of propagation and average velocity of the beams, as well as the anisotropy of their velocity field. Simple glyphs look like a quiver of arrows with a common origin located in the cell in question. The size of each arrow is set in proportion to the mean speed of the corresponding cluster so that the glyphs, on average, fit into the space allotted to them in the figure.

Composite glyphs include a quiver of arrows with a common origin and a set of ellipses with a common center located in the cell in question. The dimensions and orientation of the ellipse for each cluster are calculated from the parameters of the SD-tensor by the Formula (8) taken for the case N=2.

**Figure 7:**
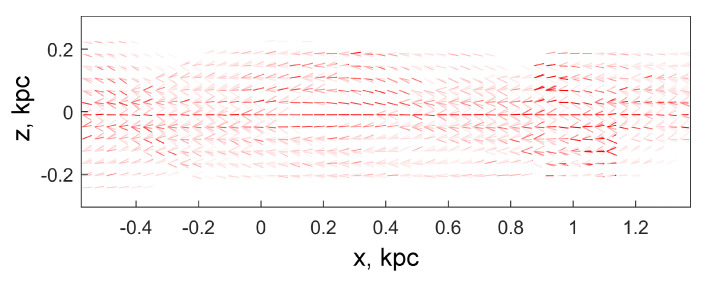
Fields of glyphs of the mean velocities of beams for a large fraction of particles. All glyphs are built for areas of 0.024×0.024 kpc (6×6 cells). The arrow lengths are proportional to the mean velocity of the beam particles. The color saturation of glyphs characterizes the number of particles in a particular beam according to the Formula (11). Areas with a single beam are marked with a single arrow. In areas of multi-stream flow, several glyphs are displayed simultaneously, characterizing each individual beam.

**Figure 8:**
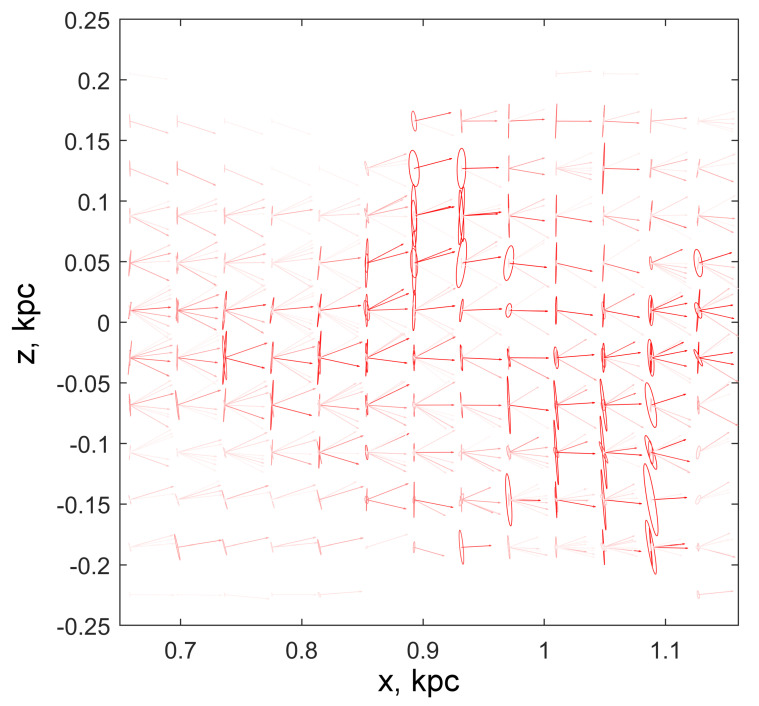
Fragment of the field of composite glyphs for beams of a large fraction of particles. All glyphs are built for areas of 0.024×0.024 kpc (6×6 cells). Each beam corresponds to a pair of objects in the glyph, an arrow and an ellipse. Arrow parameters and glyph colors are defined similarly to Figure 7. The ellipse determines the spread of the particle velocities in the beam: the semi-major axes are proportional to the standard deviations, and the directions of the ellipse axes are set by the principal axes of the tensor.

The color of the glyph is selected according to the type of particles, red, green, or blue. The intensity of the hue of a glyph color depends on the number of particles in the cluster to which it is associated: the more particles, the richer the hue. In particular, when coloring glyphs for the coarse fraction (red) in Figure 7 and Figure 8 color in palette [r,g,b] was set as follows

(11) $$r\left(n_{k}\right)=1,\;g\left(n_{k}\right)=b\left(n_{k}\right)=1-\tanh\left(n_{k}/N_{c}\right),$$

where $N_{c}=100$ is the typical cluster volume in the sample, and $n_{k}$ is the volume of the *k*-th cluster.

When constructing Figure 7 and Figure 8, the glyphs were drawn for boxes with a size of 6×6 cells so that the number of particles falling into them was, on average, sufficient for cluster analysis. The family of glyphs allows you to see directly those areas where multi-flow occurs and to evaluate its kinematic characteristics. The most powerful beams containing the largest number of particles are easily traced from the brightest arrows and ellipses.

## 8. Conclusions

In this paper, we considered the procedures for visualizing the flows of collisionless media in the form of impurities of particles immersed in the carrier gas phase. These allowed the study of the relative motion of the dispersed and carrier phases, and the uncoordinated motion of different fractions of the impurity itself, when the behavior of different types of particles differs significantly from each other.

The proposed methods were based on the analysis of the kinematic characteristics of the flow, the vector field of particle velocities, and the tensor field of velocity dispersion. When visualizing these, fields of scalar quantities may be formed, with the so-called, surrogate entropy, or eccentricity of the dispersion tensor, or fields of glyphs, which are an improvement of the usual velocity fields in the form of arrows. In the case of scalar visualization, the expressive means was color, which served as an indicator of the magnitude of the spread of velocities in a polydisperse medium.

In the case of visualization with glyphs, the kinematic structure of the flow was described both by means of color, which carried information regarding the number of particles in the beam, and by means of graphical primitives, which simultaneously combined information for both the average velocities of the beams and the spread of particle velocities in a separate beam. Using these procedures, it was possible to visualize, with the required degree of detail, the structure of the anisotropic field of velocities of a moving stream of particles, which greatly simplifies the qualitative analysis of multi-stream flows.

## 9. Patents

The tricolor technique for visualization of the hydrodynamic jumps by using the rate-of-strain tensor is protected by the Russian Federation Certificate [Korolev V.V. The program for the hydrodynamic flow structure analysis by using the rate-of-strain tensor.—RF Certificate of the Computer Program State Registration N 2012615602, 20 June 2012].
